# Early experiences with quality-assured HbA1c and professional glucose point-of-care testing in general practice: a cross-sectional observational study among patients, nurses and doctors

**DOI:** 10.1186/s12912-022-00969-0

**Published:** 2022-07-08

**Authors:** Marleen Smits, Rogier Hopstaken, Lusan Terhaag, Gijs de Kort, Paul Giesen

**Affiliations:** 1 Scientific Center for Quality of Healthcare (IQ Healthcare), Radboud University Medical Center, P.O Box 9101, 160 IQ healthcare, HB 6500 Nijmegen, The Netherlands; 2 Star-shl Diagnostic Centers, Etten-Leur, the Netherlands; 3 General Practice Hapert en Hoogeloon, Hapert, the Netherlands

**Keywords:** Primary Health Care (MeSH), Primary care nursing (MeSH), Point-of-Care Testing (MeSH), Diabetes Mellitus (MeSH), General practice

## Abstract

**Background:**

Point-of-care testing (POCT) is increasingly used in primary care. The rapid availability of the test result during the patient encounter increases the potential for patients and care providers to make a direct and joint decision on disease management. Our aim was to get insight into the first experiences of patients and healthcare professionals after introducing quality-controlled HbA1c and professional glucose POCT in diabetes care in their own general practices.

**Methods:**

A cross-sectional observational study using paper questionnaires for patients, nurses and general practitioners (GPs) in 13 general practices in the Netherlands. HbA1c and professional glucose POCT was introduced after training and under day-to-day quality control. Patients filled in the questionnaire immediately after the test; nurses and GPs after a minimum period of three months from the starting date. Descriptive data analyses were performed.

**Results:**

A total of 1551 fingerstick blood POC tests were performed (1126 HbA1c; 425 Glucose). For HbA1c POCT, 84 patients, 29 nurses and 11 GPs filled in the questionnaires. For professional glucose POCT, 30 patients, 17 nurses and 8 GPs responded. Response rates varied between 24 and 56%. Patients, nurses and GPs were generally (very) satisfied with the novel POC tests. Patients were most positive about the location (in the GPs’ office) and execution of the POC test (by their own nurse), and the speed of the test result. Almost all nurses indicated to have sufficient knowledge and skills to perform the test. Both nurses and GPs had confidence in the test results and indicated they experienced a higher patient satisfaction than with regular blood tests. Perceived disadvantages were the time required to regularly calibrate the devices and the extension of the consultation time because of the test. Patients, nurses and GPs generally expressed they wanted to continue performing these POC tests in routine diabetes care.

**Conclusions:**

Patients, nurses and GPs expressed (very) positive first experiences after introducing HbA1c and professional glucose testing on two high-quality POCT devices in their own general practices. Further research, with a random selection procedure of practices and patients and in other regions and countries, is recommended to confirm these findings.

**Supplementary Information:**

The online version contains supplementary material available at 10.1186/s12912-022-00969-0.

## Background

To guide the diagnostic process, general practitioners (GPs) increasingly ask for point-of-care testing (POCT) solutions, as a replacement for sending patients to an external laboratory or hospital to perform blood test [1]. POCT is defined in many ways, but is mostly used for laboratory testing to support clinical decision making, which is performed by a qualified member of the practice staff nearby the patient, during or very close to the time of consultation, to help the patient and physician to decide upon the best suited approach, and of which the results should be known at the time of the clinical decision making [2].

The rapid availability of the test result during the patient encounter increases the potential for patients and healthcare providers to start using POCT in their practice [3–5]. Moreover, the fingerstick test procedure is less burdensome for patients than a venepuncture and patients do not have to travel to an external hospital laboratory. Patients and healthcare professionals are generally satisfied with POCT [6, 7].

In diabetes care, glucose is routinely measured for early detection and monitoring of patients with diabetes. Although there are high-quality, professional POCT devices [8–10], most primary care professionals still use less reliable consumer tests that are meant for individual monitoring, with accepted 15% variation to reference test results. In addition to glucose, glycated haemoglobin A1c (HbA1c) POCT can contribute to better prevention, detection, and monitoring of the disease and possible complications. HbA1c POCT is not common in Dutch general practices, but it has shown to result in clinical effectiveness in diabetes care in Australian general practices [11]. HbA1c POCT has also been found to improve testing adherence compared to regular laboratory testing [12]. Reliable and user-friendly HbA1c POCT can be done with a few different POCT devices [13, 14].

Primary care data on experiences with professional glucose POCT and HbA1c POCT are lacking. In a preliminary survey among 80 primary care nurses 95% indicated they wanted to start using POCT for HbA1c, and 85% also wanted to start with professional glucose testing in the Netherlands. In another preliminary study, 64 patients with diabetes were repeatedly tested on HbA1c using the fingerstick method on Afinion 2 (Abbott) during and after a 20-weeks walking event. Of these patients, 76% wanted the HbA1c POCT measurement to be introduced in their general practices (21% neutral, 3% disagreed) [15].

Published scientific evaluations of POCT are scarce and most only describe analytical performance and user-friendliness. We studied the practical implementation and the perceived added value of POCT in general practices that received quality assurance by a diagnostic center that routinely offers a POCT service to over 1.000 GPs (see Supplemental file 1). Our aim was to get insight into the first experiences of patients, nurses and GPs with high-quality POCT for diabetes care in general practices**.**

## Methods

### Design

The study was a cross-sectional observational study with questionnaires among patients, nurses and GPs from general practices who started to use HbA1c POCT (Afinion 2, Abbott) and/or professional glucose POCT (Accu-Chek Inform II, Roche). The study was part of a larger study, that also assessed POCT not related to diabetes care (e.g. Hb, urine analyser).

### Setting and population

A selection of general practices in the southwest region of the Netherlands was approached for participation by Star-shl diagnostic centers. Star-shl is a non-commercial diagnostic center, raised by GPs. The diagnostic center innovates in line with the diagnostic wishes of the GPs. For this early experiences study GP practices were therefore only recruited, when at least one of the GPs in a GP practice had actively requested extension of the existing POCT service with HbA1c and/or professional glucose testing. All of these motivated practices were recruited, and they all participated actively in the study.

A total of 13 general practices participated: 12 in HbA1c POCT and 10 in professional glucose POCT. The practices using HbA1c POCT had 52 nurses (practice nurses and GP assistants) and 44 GPs. The practices using professional glucose POCT had 52 nurses and 34 GPs. All nurses and GPs were asked to share their experiences in a questionnaire. In addition, tested patients were asked to fill-out a questionnaire. Participants were informed about the aim of the study, privacy aspects and the involved organisations on the first page of the questionnaire. They could freely choose not to fill in the questionnaire. Exclusion applied to patients who did not have Dutch language skills.

### Questionnaire

For both HbA1c and glucose three different questionnaires were developed, i.e. for GPs, nurses and patients. The questions were based on the Measuring Instrument for Determinants of Innovations (MIDI) [16] and literature [17, 18], and were complemented with self-developed questions. The questionnaires also contained background questions and statements about POCT, which could be scored using a five-point Likert scale: ‘Strongly disagree’, ‘Disagree’, ‘Neutral’, ‘Agree’ and ‘Strongly agree’. Patients had an additional option, namely ‘Don't know/N.A.’. The questions were followed by a question whether or not the respondent would recommend the POC test to other patients who come to the general practice, on a scale from 1 ('Definitely not') to 10 ('Definitely yes'), from which the Net Promoter Score (NPS) could be calculated [19]. At the end of the questionnaires, there was room for comments. The questionnaires for professionals were checked by two GPs, two medical students, and two nurses. The questions for patients (HbA1c version) were already tested in a previous study [15].

### Data collection

The study was performed between June 2020 and May 2021. The data collection period for the patient questionnaire varied from three to eight months, depending on the starting date of the new POCT application in the general practices. The first practices started in June 2020 and the last practices in November 2020. Nurses were trained in the procedures for use of the equipment by Star-shl. Patients that underwent a POC test received a paper questionnaire from a practice employee immediately after the test, and filled this out on the site.

After a minimum period of three months from the starting date, nurses and GPs completed one questionnaire per type of POC test. Initially, they received paper questionnaires from the researchers. In the last month of the study, healthcare professionals were offered the option to complete the questionnaire(s) online (using LimeSurvey, version 2.06), if they had not already done so on paper. In addition, from the period June 2020—February 2021, data were extracted from the registration system of the diagnostic center to examine the total number of tests performed.

### Statistical analyses

We performed descriptive data analyses using IBM SPSS Statistics, version 27. When analysing the responses on the statements in the questionnaire, the answer option 'Don't know/N.A.' was considered missing data. The percentage of 'Don't know/N.A.' varied from 0 to 5% per statement. The free text comments were categorised in themes and summarised.

To assess users' satisfaction, the NPS (European version), was calculated for whether the respondent would recommend the POC test to others. The NPS is established by subtracting the percentage of detractors (score ≤ 5) from the percentage of promoters (score ≥ 8). This results in a NPS score between -100 and + 100; a higher score is desirable [19].

## Results

### Respondents

The total number of POC tests performed was 1551 (1126 HbA1c and 425 professional glucose tests). The number of patients that responded to the HbA1c questionnaire was *N* = 80, with a mean age of 64.6 years (SD 9.1; range 34–81). The number of patient respondents for the glucose questionnaire was *N* = 24. Their mean age was 59.5 years (SD 16.8; range 21–79) (Table 1).

**Table 1:** Characteristics of respondents

Characteristic	HbA1c	Professional glucose
	*N* (%)	*N* (%)
***Patients***	*N* = *80*	*N* = *24*
Gender		
Male	42 (53)	10 (42)
Age		
< 18	-	-
18–44	1 (1)	5 (21)
45–64	33 (41)	5 (21)
65–74	36 (45)	11 (46)
75 +	10 (13)	3 (12)
***Nurses***	*N* = *29*	*N* = *17*
Educational background		
Practice nurse	14 (48)	5 (63)
GP assistant	15 (52)	3 (37)
Years of experience with fingerstick blood sampling *mean (SD)*	12.9 (11.5)	8.3 (9.2)
Number of POC tests performed		
< 5	10 (36)	-
6–10	-	2 (12)
11–15	3 (11)	4 (24)
16–20	1 (4)	1 (6)
> 20	14 (50)	5 (29)

*SD* Standard deviation

No background data available from general practitioners

Twenty-nine nurses responded to the HbA1c POCT questionnaire (*N* = 29; response rate 56%) and 17 nurses to the glucose POCT questionnaire (*N* = 17; response rate 33%). The mean number of years of experience with fingerstick blood sampling was 12.9 years (SD 11.5) for HbA1c POCT users and 8.3 years (SD 9.2) for glucose POCT users. Most nurses had performed more than 20 HbA1c and/or glucose POCT measurements (Table 1). Eleven GPs responded to the HbA1c POCT questionnaire (*N *= 11; response rate 25%) and eight to the glucose POCT questionnaire (*N* = 8; response rate 24%).

### Patient experiences

Almost all patients answered positively to the statements in the questionnaires. The percentage of positive answers varied from 87 to 100%. Patients were most positive about the location (HbA1c 96%; glucose 100%) and execution (both 100%) of the POC tests, the information provided (HbA1c 99%; glucose 100%), the speed (HbA1c 96%; glucose 100%), and confidence in the reliability (HbA1c 98%; glucose 100%) of the test result (Table 2).

**Table 2:** Patient experiences with HbA1c POCT (*n* = 84) and professional glucose POCT (*n* = 30): percentage of positive answers*

Item	HbA1c	Professional glucose
	*n* (%)	*n* (%)
The GP/nurse gave me clear information about the blood test	82 (99)	30 (100)
I am satisfied with the way the blood test was performed	84 (100)	30 (100)
I prefer this fingerstick blood sampling than the regular elbow blood sampling	78 (93)	26 (87)
I am satisfied with the speed of the blood test result	81 (96)	30 (100)
I have confidence in the blood test result	82 (98)	30 (100)
I like that I do not have to go to the laboratory to perform the test	81 (96)	30 (100)
I experienced the blood test as burdensome**	79 (94)	29 (97)
I want my general practice to continue with this way of blood testing	79 (98)	30 (100)

^*^Positive answers: answer option 4 ('agree') or 5 ('strongly agree')

^**^ This item was reverse coded: a positive answer means that the patient did not experience it as burdensome

**Fig. 1:**
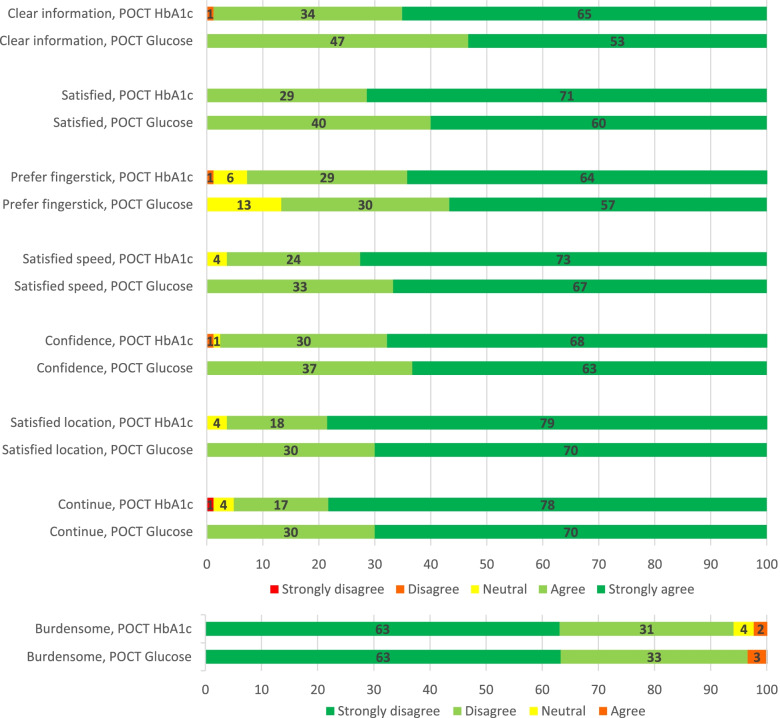
Patient experiences with HbA1c POCT (*n* = 84) and professional glucose POCT (*n* = 30): percentages on total answering scale

Figure 1 shows the percentages of responses per item on the total scale of answering categories. For HbA1c POCT, one and the same patient disagreed with the statements about the information given, preference for fingerstick, confidence in the result and continuation of the POCT blood test. Two patients experienced the HbA1c blood test as burdensome and one patient experienced the glucose blood test as burdensome. For both types of POCT, all other patients agreed. The percentage of respondents who 'strongly agreed' with a statement was highest for the statements “I like that I do not have to go to the laboratory to perform the test” (HbA1c 79%; glucose 70% strongly agree) and “I want my general practice to continue with this way of blood testing” (HbA1c 78%; glucose 70% strongly agree) (Fig. 1).

Patients were asked whether they would recommend the POCT study to other patients, giving a number from 1 to 10 (1 = 'definitely not'; 10 = 'definitely yes'). The mean recommendation rate for HbA1c POCT was 9.0 (SD 1.1; range 6–10). The mean recommendation rate for professional glucose POCT was 8.8 (SD 1.1; range 7–10). Both types of POCT scored an NPS of 86.

### Experiences of healthcare professionals

Almost all nurses answered positively to the questions “The measurement corresponds with the way I am used to work” (HbA1c 86%, glucose 94%), “I have sufficient knowledge to perform the measurement” (HbA1c 97%, glucose 100%), "I have sufficient skills to perform the measurement" (HbA1c 97%, glucose 94%) and "I have confidence in the result of the measurement" (HbA1c 97%, glucose 100%). Statements about which they were least positive are “I would like to receive feedback about my error messages when performing the measurement” (HbA1c 54%, glucose 59%) and “The measurement increases my work satisfaction” (HbA1c 62%, glucose 59%). More than three quarters of the nurses wanted their practice to continue with POCT (HbA1c 83%, glucose 76%) (Table 3).

**Table 3:** Experiences of healthcare professionals with HbA1c POCT and professional glucose POCT: percentage of positive answers*

Item	HbA1c		Professional glucose	
	**Nurses *n* = 29 **n (%)	**GPs *n* = 11 **n (%)	**Nurses *n* = 17 **n (%)	**GPs *n* = 8** n (%)
The measurementˠ corresponds with the way I am used to work	25 (86)	N/A	16 (94)	N/A
I have sufficient knowledge to perform the measurementˠ	28 (97)	N/A	17 (100)	N/A
I have sufficient skills to perform the measurementˠ	28 (97)	N/A	16 (94)	N/A
I spend little time on the measurementˠ	22 (76)	N/A	11 (65)	N/A
I can operate the device easily	27 (93)	N/A	15 (88)	N/A
I have confidence in the results of the measurementˠ	28 (97)	11 (100)	17 (100)	7 (88)
Patients are generally satisfied with the measurementˠ	26 (90)	9 (82)	14 (82)	7 (88)
The measurementˠ contributes to better patient care	22 (76)	9 (82)	14 (82)	4 (50)
The measurementˠ increases my work satisfaction	18 (62)	6 (55)	10 (59)	4 (50)
The measurementˠ is an improvement over the usual situation	19 (66)	7 (70)	12 (71)	5 (63)
The support from Star-shl diagnostic centers with regard to the measurementˠ is good	18 (62)	5 (50)	11 (65)	6 (63)
This support from Star-shl contributes to better patient care	20 (69)	6 (55)	12 (71)	5 (75)
I would like (nurses) to receive feedback about my (their) error messages when performing the measurementˠ	15 (54)	8 (80)	10 (59)	5 (63)
I want our practice to continue the measurementˠ in collaboration with the supporting diagnostic center (Star-shl)	24 (83)	9 (82)	13 (76)	4 (50)

^*^Positive answers: answer option 4 ('agree') or 5 ('strongly agree')

ˠ The questionnaires stated here “(new method) HbA1c measurement” or “professional glucose measurement”

*N/A* Not applicable

GPs gave the highest scores for their confidence in the result of the measurement (HbA1c 100%, glucose 88%) and patient satisfaction (HbA1c 82%, glucose 88%). They were least positive about the statement “The measurement increases my work satisfaction” (HbA1c 55%, glucose 50%). Of the GPs, 82% wanted their practice to continue with HbA1c POCT and 50% with glucose (Table 3).

**Fig. 2:**
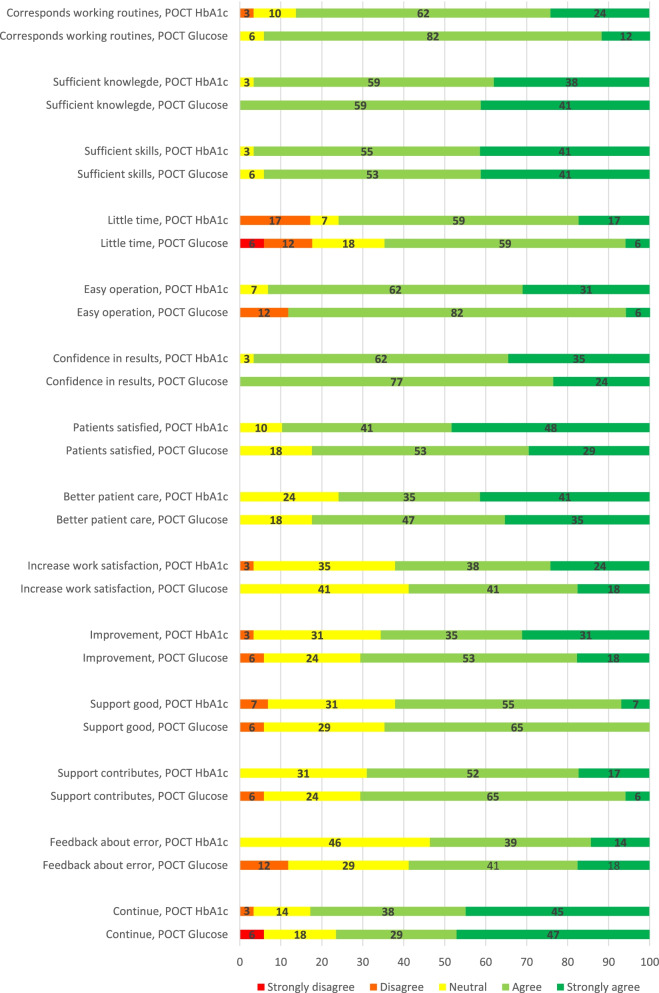
Nurses’ experiences with HbA1c POCT (*n* = 29) and professional glucose POCT (*n* = 17): percentages on total answering scale

Figure 2 shows the responses of the nurses per item on the total scale of answering categories. The percentage of nurses that disagreed with a statement was largest for “I spend little time on the measurement” (HbA1c 17% (*n* = 5); glucose 18% (*n* = 3)). Furthermore, more than one respondent gave a negative response to the statements related to the operation of the device (glucose 12% (*n* = 2)) and desire for feedback on error (glucose 12% (*n *= 2)).

Nurses recommended HbA1c POCT to other general practices with a mean score of 8.6 (SD 1.5; range 4–10) and GPs with a mean score of 7.6 (SD 1.4; range 5–9). The NPS of HbA1c POCT was 76 for nurses and 55 for GPs. The mean recommendation score of professional glucose POCT was 8.1 for nurses (SD 1.3; range 5–10) and 6.6 for GPs (SD 1.5; range 5–8). The NPS of professional glucose POCT was 75 for nurses and 25 for GPs.

In the free text boxes healthcare professionals most often cited the speed of the test result as an advantage of POCT. They mentioned that the test result is already available during the encounter and, therefore, they can give advice and start treatment immediately. In addition, while waiting for the test results, nurses have different kinds of conversations with patients and they get to know them better. Other advantages are that the results of the tests are stored automatically in the patient records, that patients are happy they do not have to go to another location (blood sampling service of the diagnostic center) for the test, and that with POCT they catch people with a fear of needles who normally do not get tested on a regular basis.

However, POCT also involves an investment of time for the general practices. Nurses specified that the consultation time is extended by the actions required to perform the test (scanning, preparing things, starting up, etc.) and by waiting for the test results. In addition, they indicated that they experience the weekly checks of the equipment as time-consuming and burdensome in relation to the frequency with which the equipment is used.

## Discussion

### Main findings

We examined the first experiences of patients, nurses and GPs with HbA1c and professional glucose POCT in their own general practices as newly added POCT services of a diagnostic center. More than 1500 tests in 13 general practices were performed. All items in the questionnaires were scored positively by the majority of the respondents. For patients, the speed and the location (GP’s office) of POCT seem to be important improvements over regular blood testing**.** Nurses and GPs seem to have sufficient knowledge and skills, to experience high patient satisfaction and to have confidence in the test results. There was no apparent improvement in job satisfaction, nor a common desire to receive feedback about error messages when performing the measurement. The extra workload seems to be the only disadvantageous factor: time is needed to prepare the measurement and to wait for the result during the consultation. Extra time is also needed to calibrate the equipment regularly. Patients, nurses and GPs generally are in favour of continuing the POC tests in their offices. The Net Promotor Scores (NPS) for both types of POCT were positive.

### Comparison with other studies

Various studies have reported on experiences with POCT. Some of these studies were performed in the primary care setting. Primary care patients preferred POCT in their own general practice over regular blood collection in the laboratory, with the major advantages being the rapid test result, less invasive test procedure and the location of the test [6, 15, 20]. These findings were confirmed by our study. Specifically for HbA1c POCT, studies have shown that the immediate availability of the results is motivating for patients and leads to better diabetes control [21, 22].

A worldwide survey of POCT analysts (mostly nurses) from both general practices and hospitals showed that they believed POCT contributes positively to patients’ healthcare and safety [23], which corresponds with our results. However, most POCT analysts desired more training and quality checks [23], whereas the nurses in our study were satisfied with the training and support from the diagnostic center, and reported they had to spend much time on checks of the devices. Specifically for HbA1c POCT, a qualitative study reported the main advantage for nurses was having the result available immediately for discussion with the patient, which was also mentioned by the nurses we interviewed [24].

A systematic review into primary care clinicians’ (mainly GPs) attitudes towards POCT blood testing showed that they believed that POCT improved diagnostic certainty, targeting of treatment, self-management of chronic conditions, and clinician-patient communication and relationships [7]. This is in line with our study results. However, the clinicians raised concerns about test accuracy, over-reliance on tests, undermining of clinical skills, cost, and limited usefulness [7]. These concerns were not reported by the GPs in our study.

### Strengths and limitations

As far as we know, our study is the first ever to report on the experiences with professional glucose testing, i.e. glucose POCT performed on quality-assured professional devices instead of consumer glucose strip testing. In our study, a large number of tests was performed. The ample experience with these new POCT methods improves reliability of the users’ responses. We selected only benevolent general practices in a southwestern region of the Netherlands. It is not known whether the practices are representative for other general practices in the Netherlands or abroad. We chose this selection strategy to evaluate whether POCT works in suited and motivated practices, before widespread implementation. It is not expected that patient responses would have been different with another selection procedure for the general practices.

The response rate was moderate for nurses (33–56%) and quite low for GPs (24–25%). Due to the Covid-19 pandemic, the healthcare professionals had less attention for the study, and the diagnostic center could not regularly visit the participating practices for monitoring as intended. It is not known how many patients were invited to participate. Therefore, no patient response rate could be calculated. Moreover, it is possible that participants who were very positive about POCT were more likely to complete the questionnaire. Because of the possible selection bias, the results of this study should be interpreted with caution.

### Implications for practice and further research

POCT can contribute to easier, faster and better diagnostic testing and monitoring of various illnesses. Previously, CRP POCT has been evaluated very positively and it is now being used routinely in most Dutch GP practices. The two newly introduced POCT measurements, HbA1c and professional glucose, were judged as valuable additions for better diabetes care. This primarily holds promise for nurses who intensively work with patients with diabetes in daily practice. Further research on a larger scale is recommended. In order to reduce the chance of selection bias, practices and patients should be selected randomly. Alternatively, all consecutive patients could be included in a limited time frame. Monitoring their response and reacting to it could increase the response rate.

It is recommended to make POCT more attractive and user-friendly for the nursing staff. POCT expertise teams should consider new ways to unburden GP nurses in their busy practices, for example by installing enhanced software for easier automatic monitoring, and by applying less frequent quality controls, if the nurses and practices have proven to perform well in the past (control-by indication). Moreover, user-friendly POC devices with logic test panels help to reduce variation of POCT testing and control procedures, to increase patient safety, and to improve testing performance by the users.

## Conclusions

Patients, nurses and GPs are satisfied with newly introduced POCT methods for diabetes care. They would like to continue using them in their general practices. A barrier is the possible extra workload for nurses, especially due to the quality assurance procedures. Further research on a larger scale and with a random selection of practices and patients is needed.

## Supplementary Information

**Additional file 1.**

## Data Availability

The datasets generated during and analyzed during the current study are not publicly available due to Dutch language but are available from the corresponding author on reasonable request.

## Abbreviations

GP: General practitioner
HbA1c: Glycated haemoglobin A1c
N/A: Not applicable
POCT: Point-of-care testing
SD: Standard deviation
SPSS: Statistical Package for the Social Sciences
