# Supplementary material for: Early experiences with quality-assured HbA1c and professional glucose point-of-care testing in general practice: a cross-sectional observational study among patients, nurses and doctors
Source: BMC Nurs. 2022 Jul 8;21:183. doi: 10.1186/s12912-022-00969-0 (PMC9263435; doi:10.1186/s12912-022-00969-0)
Supplement: Supplementary file 1 — Additional file 1. [file 12912_2022_969_MOESM1_ESM.docx]

**Supplemental file 1 - Quality assurance by diagnostic center**

The diagnostic center Star-SHL has set-up a POCT service that adheres to both the international laboratory ISO norms, as well as to the national collaborative guideline on POCT in general practice. The quality of care and safety of patients need to be ensured accordingly. In daily practice, this is executed by a dedicated team of POCT experts from the diagnostic center (clinical pathologists, POCT analysts, POCT specialists) who routinely check and monitor the quality of the POCT service in- and outside the diagnostic center. Main elements are: a) critical appraisal and/or performance of scientific studies on the clinical value of a specific (new) test; b) validation and verification studies on performance of each new device and its specific assays; c) technical and test performance checks per device and per lot of tests; d) location checks for the suitability of the exact place where POCT will be performed; e) instruction, training and accreditation of all identified users in general practice; f) ongoing (24/7) checks on the performance of the users and devices, both physically in the practice and remotely via installed communication interfaces to detect errors; g) acting on technical and user errors as soon as possible to maintain high quality and safety of patient care; h) logistics and organisational flow of the POCT service.
